# An Increase in Liver Polyamine Concentration Contributes to the Tryptophan-Induced Acute Stimulation of Rat Hepatic Protein Synthesis

**DOI:** 10.3390/nu12092665

**Published:** 2020-09-01

**Authors:** Shinichiro Koike, Yukihito Kabuyama, Kodwo Amuzuah Obeng, Kunio Sugahara, Yusuke Sato, Fumiaki Yoshizawa

**Affiliations:** 1 Department of Biological Production Science, United Graduate School of Agricultural Science, Tokyo University of Agriculture and Technology, Fuchu, Tokyo 183-8509, Japan; shinichiro@UCDAVIS.EDU (S.K.); kabuyama@cc.utsunomiya-u.ac.jp (Y.K.); s167797r@st.go.tuat.ac.jp (K.A.O.); sugawara@cc.utsunomiya-u.ac.jp (K.S.); ysato@cc.utsunomiya-u.ac.jp (Y.S.); 2 Department of Applied Biological Chemistry, School of Agriculture, Utsunomiya University, Utsunomiya, Tochigi 321-8505, Japan; 3 Department of Agrobiology and Bioresources, School of Agriculture, Utsunomiya University, Utsunomiya, Tochigi 321-8505, Japan

**Keywords:** tryptophan, protein synthesis, omics, liver, rat

## Abstract

Tryptophan has a unique role as a nutritional signaling molecule that regulates protein synthesis in mouse and rat liver. However, the mechanism underlying the stimulating actions of tryptophan on hepatic protein synthesis remains unclear. Proteomic and metabolomic analyses were performed to identify candidate proteins and metabolites likely to play a role in the stimulation of protein synthesis by tryptophan. Overnight-fasted rats were orally administered L-tryptophan and then sacrificed 1 or 3 h after administration. Four differentially expressed protein spots were detected in rat liver at 3 h after tryptophan administration, of which one was identified as an ornithine aminotransferase (OAT) precursor. OAT is the main catabolic enzyme for ornithine, and its expression was significantly decreased by tryptophan administration. The concentration of ornithine was increased in the liver at 3 h after tryptophan administration. Ornithine is a precursor for polyamine biosynthesis. Significantly increased concentrations of polyamines were found in the liver at 3 h after administration of tryptophan. Additionally, enhanced hepatic protein synthesis was demonstrated by oral administration of putrescine. We speculate that the increase in ornithine level through suppression of OAT expression by tryptophan administration may lead to accelerated polyamine synthesis, thereby promoting protein synthesis in the liver.

## 1. Introduction

Tryptophan (Trp) is an essential amino acid that cannot be synthesized in the body and must be supplied by the diet [1,2]. The principal role of Trp is a constituent of proteins. Of the 20 amino acids that comprise proteins, Trp is present in the lowest proportion in both proteins and plasma [3]. Therefore, Trp is relatively less available than the other amino acids and is thought to play a rate-limiting role during protein synthesis [1]. Furthermore, it has been reported that Trp has a unique role as a nutritional signaling molecule that regulates protein synthesis in mouse and rat liver, in addition to its importance as a constituent of protein. Sidransky et al. demonstrated that a single tube feeding of Trp to fasted animals (mice or rats) rapidly shifted hepatic polyribosomes toward heavier aggregation states and increased hepatic protein synthesis as measured in vivo and in vitro [4,5]. Subsequently, they reported a Trp receptor in the hepatic nuclear envelope, and that the binding of Trp to this receptor is saturable, stereospecific, and of high affinity [6]. They speculated that this specific binding of Trp to hepatic nuclei plays a vital role in the ability of Trp to rapidly stimulate hepatic protein synthesis [7,8]. However, the mechanism underlying the stimulating actions of Trp on hepatic protein synthesis has not been clearly elucidated.

Trp is also a precursor for a variety of biologically active compounds, including serotonin, melatonin, tryptamine, quinolinic acid, and kynurenic acid. In addition, Trp is a precursor for the coenzymes nicotinamide adenine dinucleotide (NAD) and nicotinamide adenine dinucleotide phosphate (NADP), and can replace niacin as an essential nutrient. Several recent studies highlight the importance of serotonin in liver regeneration after partial hepatectomy [9]. Treatment with L-Trp increased hepatic mammalian target of rapamycin (mTOR) phosphorylation after food deprivation, and serotonin production was crucial for Trp-mediated mTOR phosphorylation in the liver [10]. The phosphorylation of mTOR leads to increased protein synthesis via phosphorylation of downstream targets such as eukaryotic initiation factor 4E binding protein 1 (4E-BP1) and ribosomal protein S6 kinase (S6K), leading to cell growth and proliferation. Therefore, we cannot exclude the possibility that Trp metabolites contribute to the Trp-induced stimulation of hepatic protein synthesis.

Recently, “omics” technologies such as genomics, proteomics, and metabolomics have become widely popular as complementary technologies for conventional analytical methodologies. These powerful discovery tools allow studies of the functions of amino acids and enable in-depth investigation of the regulatory roles of dietary amino acids as a nutritional signaling molecule in gene and protein expression. Trp has been suggested to stimulate protein synthesis at the translational level because polysome aggregation was observed shortly after oral administration of Trp to rats [4]. Therefore, we focused on the acute effects of Trp as a nutritional signaling molecule on protein synthesis. In the present study, proteomic and metabolomic analyses were performed to identify likely candidate proteins and metabolites that play a role in the stimulation of protein synthesis by Trp.

## 2. Materials and Methods

### 2.1. Animals and Experimental Design

The animal care protocol for this experiment was approved by the Utsunomiya University Animal Research Committee under the Guidelines for Animal Experiments of Utsunomiya University (approval number: A10-0014, approval date: 22 April, 2010). Three-week-old male Wistar rats purchased from Clea Japan (Tokyo, Japan) were individually housed in stainless steel wire cages and maintained at 22 °C under a 12-h light-dark cycle (lights on from 6:00–18:00). They were allowed free access to water and AIN-93G [11] standard diet for 10 days, then were food-deprived for 18 h from 15:00 to 9:00 the following morning. The control group received an equivalent volume of distilled water by oral gavage as a vehicle control and sacrificed after food-deprivation. The rats in the treatment groups were sacrificed after 1 or 3 h following oral gavage of L-Trp or putrescine (Put). L-Trp was suspended in distilled water and administrated at 135 mg/100 g bw. The amount of Trp administered and experimental design were determined based on a previous study, which indicated that the single oral administration of 135 mg/100 g bw of leucine was sufficient to stimulate protein synthesis in rat skeletal muscle by accelerating translation initiation [12,13,14]. This amount of Trp is equivalent to 7–10 times the amount of Trp consumed in a 24-h period by age- and strain-matched rats allowed free access to the AIN-93G standard diet. Put was similarly suspended in distilled water and 8.4 mg (1/16), 16.9 mg (1/8), or 33.8 mg/100 g bw (1/4) was administered. The amount of Put corresponded to 1/16, 1/8, and 1/4 of Trp administered (135 mg/100 g bw). The number of animals used in each experiment is indicated in the figure legends.

### 2.2. Sample Collection

The rats were killed by decapitation. Serum was obtained from the trunk blood for measurement of amino acid concentrations using an automatic amino acid analyzer (JLC500-V2, JEOL Ltd., Tokyo, Japan) after sulfosalicylic acid treatment. Immediately after the blood sample was taken, the liver was excised and weighed. For proteomic analysis, the collected liver was cut into small pieces with scissors in an ice-cold phosphate buffered saline. After centrifugation at 1000× *g* for 10 min at 4 °C, the resulting pellet was homogenized in four volumes of an ice-cold homogenization buffer consisting of 7 M urea, 2 M thio-urea, 4% CHAPS, and 25 mM Tris-HCl. Homogenates were centrifuged at 100,000× *g* for 20 min at 4 °C and the supernatants were stored at −80 °C until proteomic analysis was performed. The remaining portion of the liver was homogenized in seven volumes of an ice-cold homogenization buffer consisting of 20 mM *N*-2-hydroxyethylpiperazine-*N*′-2-ethanesulfonic acid (pH 7.4), 100 mM KCl, 0.2 mM EDTA, 2 mM ethylene glycol-bis (β-aminoethyl ether)-*N*,*N*,*N*′,*N*′-tetraacetic acid, 1 mM dithiothreitol, 50 mM NaF, 50 mM β-sodium glycerophosphate, 0.1 mM phenylmethylsulfonyl fluoride, 1 mM benzamide, and 0.5 mM sodium orthovanadate (V) using a Dounce homogenizer (Wheaton, Millville, NJ, USA). The homogenates were centrifuged at 10,000× *g* for 10 min at 4 °C. The supernatant was combined with an equal volume of 2×SDS sample buffer (2 mL of 0.5 M Tris, pH 6.8, 2 mL of glycerol, 2 mL of 10% SDS, 0.2 mL of β-mercaptoethanol, 0.4 mL of a 4% solution of bromophenol blue, and 1.4 mL of water to a final volume of 8 mL), heated for 3 min at 100 °C, and stored at −80 °C until Western blotting analysis was performed. For metabolomic analysis, the liver was collected and immediately snap-frozen using liquid nitrogen, then stored until further analysis.

### 2.3. Proteomic Analysis

#### 2.3.1. Two-Dimensional Electrophoresis

The protein concentrations of the liver extracts were determined by the Bradford method using a Bio-Rad protein assay kit (Bio-Rad Laboratories, Hercules, CA, USA). All steps of the two-dimensional electrophoresis (2-DE) analyses were conducted in pairs comprising the control and test samples. Isoelectric focusing was performed using Immobiline Dry Strip Gels, pH 3–10 (GE Healthcare, Little Chalfont, UK) in an Ettan IPGphor system (GE Healthcare). Each protein sample (500 µg) was focused at 70 kV/h with four step voltages from 500 to 8000 V. After isoelectric focusing, the gel strip was placed on a 12.5% (*w*/*v*) polyacrylamide gel (18 × 16 cm) and proteins were separated according to their molecular weight. Subsequently, the gel was stained with Coomassie Brilliant Blue (CBB) to visualize the protein spots.

#### 2.3.2. Gel Analyses

CBB-stained gels were analyzed following the methods described previously [15] using the Melanie Viewer version 3.08 software (Geneva Bioinformatics (GeneBio) SA, Geneva, Switzerland) and Image Master 2D Platinum 5.0 software (GE Healthcare). After the image analysis, protein spots whose expression changed significantly in four or more of six independent experiments were subjected to mass spectrometry.

#### 2.3.3. Mass Spectrometry

For protein identification, chosen stained protein spots in the CBB-stained gels were digested in-gel with trypsin, then analyzed by matrix-assisted laser desorption/ionization time-of-flight mass spectrometry (MALDI-TOF MS) using an Autoflex II TOF/TOF system (Bruker Daltonics GmbH, Faellanden, Switzerland). Peptide mass fingerprint spectra were matched against the National Center for Biotechnology Information (NCBI) non-redundant protein database or the Swiss-Prot protein database using the Mascot protein identification software (Matrix Science Ltd., London, UK). The mass tolerance of the fragment ions was 0.5 Da, and searches specified trypsin cleavages. The significance of each identification was evaluated by calculating the probability-based MOWSE score.

### 2.4. Immunoblotting

Proteins separated by 2-DE or SDS-PAGE were transferred to a PVDF membrane and then immunoblotted with anti-BCKDE1α (branched chain keto acid dehydrogenase E1 alpha) (#sc-271538, Santa Cruz Biotechnology, Inc. Dallas, TX, USA), anti-OAT (ornithine aminotransferase) (#sc-374243, Santa Cruz Biotechnology, Inc.), anti-ODC (ornithine decarboxylase) (#sc-33539, Santa Cruz Biotechnology, Inc.), anti-S6K1 (ribosomal protein S6 kinase beta-1) (#sc-230, Santa Cruz Biotechnology, Inc.) and anti-4E-BP1 (eukaryotic translation initiation factor 4E-binding protein 1) (#sc-6936, Santa Cruz Biotechnology, Inc.) antibodies. After incubation with appropriate secondary antibodies, the proteins were visualized using enhanced chemiluminescence (ECL, GE Healthcare). Pixel intensities of the immunoreactive bands were quantified using the Image Master 2D Platinum 5.0 software (GE Healthcare) or Image Lab software (Bio-Rad). The phosphorylation of S6K1 and 4E-BP1 was determined as described previously [12,13,14].

### 2.5. Metabolomic Analyses

Metabolome analyses were carried out by Human Metabolome Technologies Inc. (Yamagata, Japan) following methods reported previously [16,17,18]. Briefly, 100 mg of frozen liver sample was plunged into 500 μL of methanol containing 50 μM internal standards. The tissue was homogenized using a homogenizer (Shake Master NEO, BMS-M10N21 Biomedical Science, Tokyo, Japan), then the sample was mixed with 500 μL chloroform and 200 μL Milli-Q water and centrifuged at 2300× *g* at 4 °C for 5 min. Each group was pooled into one sample and 400 μL of pooled sample was filtered by centrifugation through a Millipore 5-kDa cutoff filter (Millipore-Japan, Tokyo, Japan) at 9100× *g* at 4 °C for 120 min. The filtrate was re-suspended in 50 μL Milli-Q water for CE-MS analysis. CE-TOFMS analysis was performed using an Agilent CE-TOFMS system (Agilent Technologies, Santa Clara, CA, USA). The detected peak data were analyzed using the MasterHands version 2.9.0.9 software (Keio University, Tokyo, Japan). The peaks were identified by matching m/z values and migration times with the HMT metabolite library. The tolerance for migration time and m/z were set to 0.5 min and 10 ppm, respectively. All concentrations of identified compounds were calculated by a single-point calibration with a standard compound. Redundant features, such as isotopic-, fragment-, and noise-derived peaks were removed before statistical analysis.

### 2.6. Statistical Analyses

Data are expressed as means ± standard error of the mean (SEM). Statistical significance between multiple groups were assessed by one-way analysis of variance followed by the Tukey–Kramer multiple comparisons test. Student’s t-test was used for statistical comparisons of two groups. Metabolome data comparison was performed using Welch’s t-test. Differences were considered significant at *p* < 0.05.

## 3. Results

### 3.1. Effects of Trp Administration on Serum Amino Acid Levels

The effect of Trp administration on serum amino acid concentrations was determined. At 1 h after administration of Trp, the serum concentrations of Trp (32.3-fold), taurine (Tau) (1.6-fold), aspartic acid (Asp) (6.8-fold), threonine (Thr) (1.3-fold), glutamic acid (Glu) (1.8-fold), and isoleucine (Ile) (1.3-fold) were significantly increased compared with the control group (Table 1). The serum concentrations of lysine (Lys) (0.7-fold) and arginine (Arg) (0.8-fold) decreased significantly at 1 h after Trp administration (Table 1). At 3 h after administration of Trp, Trp (26.7-fold), Tau (1.4-fold), Asp (7.7-fold), Glu (2.1-fold), glutamine (Gln) (1.4-fold), citrulline (Cit) (1.4-fold), alpha-aminobutyric acid (α-ABA) (2.2-fold), branched-chain amino acids (BCAAs) (valine (Val) (1.6-fold), leucine (Leu) (1.6-fold), Ile (1.4-fold)), ornithine (Orn) (2.1-fold) and histidine (His) (1.5-fold) levels were significantly higher than those in the control group (Table 1). The levels of glycine (Gly) (0.6-fold), Lys (0.5-fold), Arg (0.8-fold), and hydroxyproline (hypro) (0.7-fold) were significantly decreased at 3 h after Trp administration compared with the control group (Table 1).

### 3.2. Changes in the Levels of Amino Acids and Metabolites in the Liver Following Trp Administration

In the present study, 242 metabolites were identified in the liver. Three hours after Trp administration, the levels of 128 of these metabolites were significantly changed compared with the control group. In particular, the TCA cycle, the urea cycle, polyamine metabolism, kynurenine metabolism, nicotinic acid and nicotinamide metabolism, purine metabolism, and glutathione metabolism appeared to be affected by Trp administration. Of the metabolic pathways affected by Trp administration, we focused on polyamine metabolism because polyamines are believed to play important roles in protein synthesis processes. Three polyamines (Put, spermidine, and spermine) are part of the very tightly regulated polyamine metabolic pathway. The concentrations of Put (11-fold), spermidine (1.8-fold), and spermine (1.3-fold) in the liver were significantly increased compared to the control group (Table 2). Polyamines are synthesized from ornithine, a urea cycle intermediate. The concentrations of the urea cycle intermediates ornithine (1.5-fold), argininosuccinic acid (2.7-fold), and Arg (1.3-fold) were significantly increased in the Trp-administered group (Table 2). In addition, Glu (2.3-fold) and N-acetylornithine (1.8-fold), which are precursors of ornithine, were also increased in the Trp-administered group (Table 2). These results suggested that polyamine synthesis was stimulated by Trp administration. In addition, we evaluated the levels of BCAAs, which play a role in the regulation of protein metabolism in the liver [12,13,14]. The concentrations of BCAAs were significantly decreased (Val (0.8-fold), Leu (0.8-fold), Ile (0.8-fold)) after Trp administration (Table 2).

### 3.3. Changes in Protein Expression in the Liver after Oral Administration of Trp

**Figure 1:**
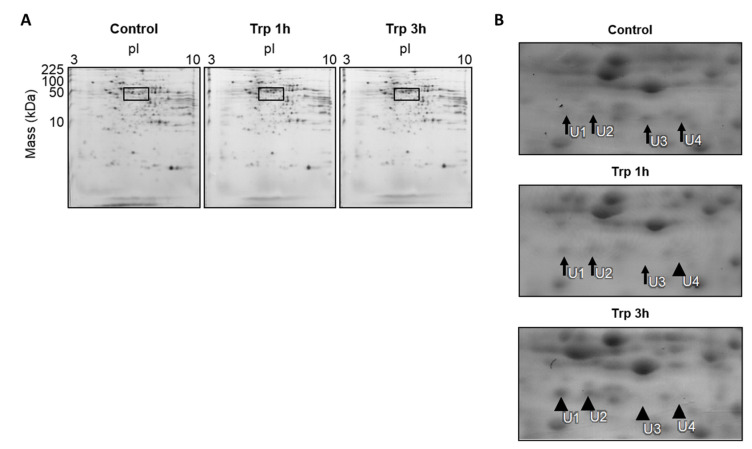
Changes in protein expression in the liver after oral administration of Trp. (**A**) Shown are representative CBB-stained 2-DE gels of whole liver lysate (250 μg of protein for pI 3–10). Control animals were sacrificed after administration of vehicle control. Trp 1h and Trp 3h were sacrificed 1 or 3 h after Trp administration (135 mg/100 g body weight), respectively. The region in each gel surrounded with a square contains protein spots differentially up- or downregulated in the Trp-administered group. (**B**) Selected regions of 2-DE gels showing the candidate protein spots U1, U2, U3, and U4. Large arrowheads highlight protein spots that were differentially up- or downregulated by Trp administration. These proteins were identified by in-gel digestion and peptide mass fingerprinting, as shown in Table 3.

Liver protein extracts were separated by 2-DE over the pI range 3–10, resolving 500 protein spots (Figure 1A). Experiments were carried out independently and repeated six times. Changes were accepted when at least four independent experiments showed a significant change in intensity. One and four protein spots changed in intensity at 1 and 3 h after Trp administration, respectively (Figure 1B). The differentially expressed protein spots were digested in-gel, then identified by peptide mass fingerprinting and confirmed by peptide sequencing using tandem mass spectrometry (Table 3).

One protein spot (spot no. U4) was significantly downregulated (0.61-fold) in the Trp 1h group (Figure 1B). This protein spot was identified as ornithine amino transferase (OAT), a mitochondrial precursor (Table 3). OAT is a key enzyme in the biosynthetic pathway of ornithine [19]. OAT catalyzes the interconversion of ornithine to glutamate and proline via the intermediate L-glutamate 5-semialdehyde, and it is the main catabolic enzyme for ornithine. OAT is synthesized in the cytoplasm as a precursor containing a leader sequence. The precursor is transported to the mitochondrial matrix where processing occurs, with cleavage of the leader sequence to form the mature subunit [20]. In the Trp 3h group, the expression of OAT was significantly decreased (0.13-fold) (Figure 1B) (Table 3). The remaining three protein spots, indicated as U1, U2, and U3, were identified as fragments of 2-oxoisovalerate dehydrogenase subunit alpha, mitochondrial (BCKDE1α). BCKD is a key enzyme in the BCAAs degradation pathway [21] (Figure 1B) (Table 3).

### 3.4. Quantification of OAT and BCKDE1α Expression

**Figure 2:**
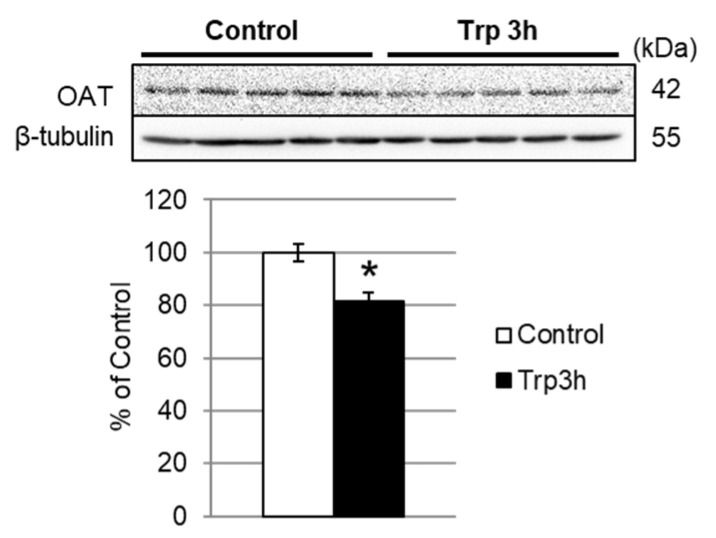
Decreased OAT protein expression in the liver after oral administration of Trp. Liver lysates were immunoblotted for OAT. The bar graph shows OAT expression normalized with β-tubulin expression. Data are expressed means ± SEM, *n* = 5. (*) Indicates significant difference between control versus Trp 3h (*p* < 0.05).

**Figure 3:**
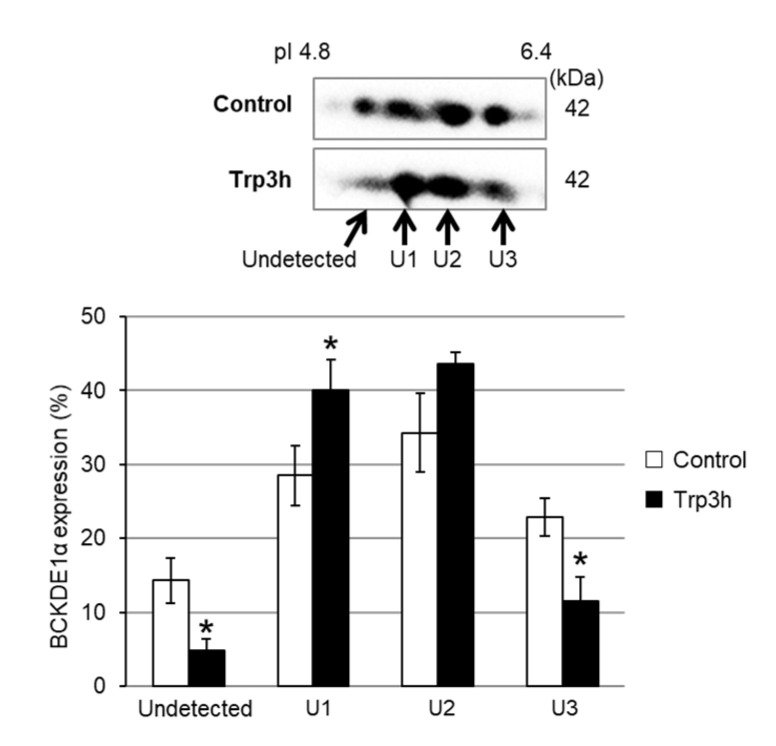
Altered expression of BCKDE1α in the liver after oral administration of Trp. Liver lysates were immunoblotted for BCKDE1α. The bar graph shows the expression of each BCKDE1α protein spot as a percentage of total BCKDE1α expression (Undetected +U1+U2+U3). Data are expressed as means ± SEM, *n* = 5. The protein spot designated “Undetected” could not be detected in CBB-stained gels. (*) Indicates significant difference between control versus Trp 3h (*p* < 0.05).

Proteomic data indicated different levels of protein expression in the control and Trp-administered groups. However, we could not exclude the possibility of false-positive findings in the proteomic analyses. To address this issue, we quantified OAT and BCKDE1α expression by Western blotting. We determined the expression of these proteins at 3 h after administration of Trp because the serum Orn and BCAAs levels were significantly changed at this point (Table 1). OAT expression was significantly decreased in the Trp 3h group compared with the control group (0.81-fold) (Figure 2). For BCKDE1α, we performed 2D Western blotting. Antibodies against BCKDE1α reacted with four protein spots with similar masses but different pI values, suggesting the presence of various post-translationally modified forms [22] (Figure 3). Based on their apparent MW and pI on 2D gels, three of the four protein spots were identified as U1, U2, and U3. The intensity of U3 was significantly decreased by Trp administration, and the intensity of U1, which has a more acidic pI, was significantly increased. These results suggest that the Trp administration induced post-translational modification of BCKDE1α accompanying an acidic pI shift (−1.1).

### 3.5. Quantification of ODC Expression

**Figure 4:**
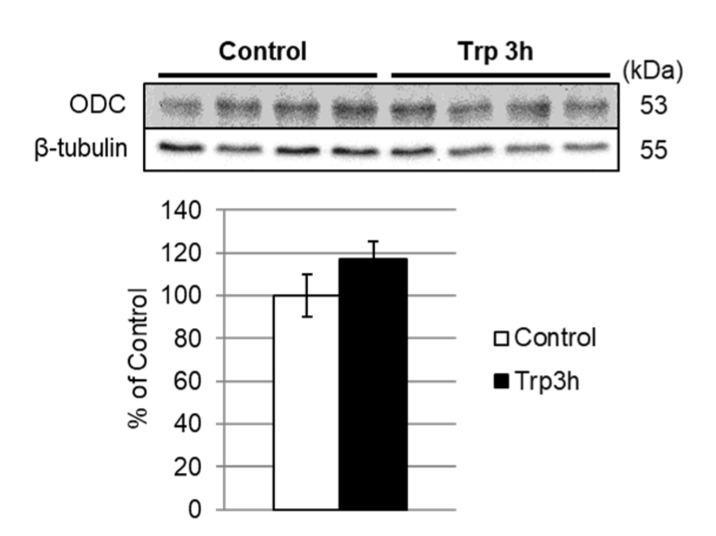
ODC (ornithine decarboxylase) expression did not alter after oral administration of Trp. Liver lysates were immunoblotted for ODC. The bar graph shows the ODC expression normalized with β-tubulin expression. Data are expressed as means ± SEM, *n* = 4.

Metabolomic analysis demonstrated that Trp administration accelerated polyamine synthesis in the liver. Polyamine biosynthesis is regulated by ornithine decarboxylase (ODC), which catalyzes the decarboxylation of Orn to form Put. Although no significant difference was observed in ODC expression by proteomic analysis, we quantitated the relative expression of ODC protein in the liver by Western blot analysis. There was no difference in the expression of ODC protein between the control and the Trp 3h group (Figure 4).

### 3.6. Effects of Putrescine Administration on Hepatic Protein Synthesis

**Figure 5:**
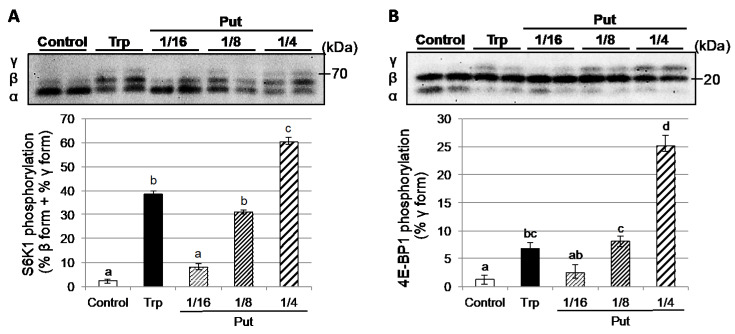
Effects of putrescine administration on hepatic protein synthesis. Liver lysates were subjected to SDS–PAGE, and the phosphorylation states of S6K1 and 4E-BP1 determined by immunoblot analysis. During SDS–PAGE, S6K1 and 4E-BP1 were resolved into multiple isoelectric forms based on its phosphorylation state. The rapidly migrating band was arbitrarily designated α, and the more slowly migrating bands as β and γ, as indicated in the blot image. (**A**) The phosphorylation of S6K1 was expressed as the amount of S6K1 in the β and γ forms as a percentage of total S6K1 (α + β + γ). (**B**) The phosphorylation of 4E-BP1 was expressed as the amount of protein present in the γ-form as a percentage of total 4E-BP1 (α + β + γ). Results represent mean ± SEM, *n* = 5. Values not sharing the same letter are significantly different (*p* < 0.05).

Polyamines (Put, spermidine, spermine) play an important role in cell proliferation and protein synthesis. It was reported that the Put treatment activated mTOR, the master regulator of protein synthesis, and its downstream targets such as S6K1 and 4E-BP1 [23]. To determine the effect of the increased liver Put level on hepatic protein synthesis, we evaluated the phosphorylation/activation of S6K1 and 4E-BP1 in the liver 3 h after the oral administration of Put. To elucidate the dose-dependent effect of Put on the liver protein synthesis, 8.4, 16.9, or 33.8 mg/100 g bw of Put was administrated. The amount of Put corresponded to 1/16, 1/8, and 1/4 of Trp administered (135 mg/100 g bw). After oral administration of 1/8 Put (16.9 mg/100 g bw) and 1/4 Put (33.8 mg/100 g bw), the phosphorylation of S6K1 and 4E-BP1 were significantly enhanced in the liver compared to the control group, but no enhancement was observed following the administration of 1/16 Put (8.4 mg/100 g bw) (Figure 5). Notably, the effect of Put administration was dose-dependent, and the effect of 1/8 Put administration was comparable to that of Trp administration.

## 4. Discussion

Sidransky et al. have reported the stimulatory effect of Trp on hepatic protein synthesis. In attempting to unravel the mechanism involved in the stimulatory response of hepatic protein synthesis by Trp, they reported that hepatic nuclei, and particularly the nuclear envelopes, have a specific receptor for L-Trp. They also suggested that the nuclear envelope receptor binding of L-Trp is important in the Trp-induced enhancement of protein synthesis. However, to date, there have been no further reports concerning the mechanism of protein synthesis stimulation by Trp.

The results of the present study suggested that an increase in polyamine synthesis could be involved in the enhancement of hepatic protein synthesis in rats, which is rapidly induced by the administration of Trp. The levels of the three polyamines Put, spermidine, and spermine were significantly increased in the liver of Trp-administered rats (Table 2). Ornithine, a substrate for polyamine biosynthesis, is derived from the amino acid arginine as part of the urea cycle. Significantly increased concentrations of ornithine were observed in both serum (Table 1) and liver (Table 2) 3 h after Trp administration. Additionally, the concentrations of three components of the urea cycle, ornithine, argininosuccinate, and arginine, were significantly increased in the Trp-administered group. The increase in ornithine concentration could be explained by reduced OAT expression in the liver. OAT is a mitochondrial matrix enzyme that controls the L-ornithine level in tissues by catalyzing the reversible interconversion between L-ornithine and L-glutamate 5-semialdehyde [19]. OAT is synthesized in the cytoplasm as a precursor containing a leader sequence. The precursor is transported to the mitochondrial matrix where processing occurs, with cleavage of the leader sequence to form the mature subunit [20]. Proteomic analysis revealed that the protein expression level of the hepatic OAT precursor was suppressed in the Trp 3h group (Figure 1B and Figure 2) (Table 3). Consistent with our proteomic results, Western blotting confirmed decreased protein levels of OAT in the liver 3 h after Trp administration (Figure 2). These results suggest that the increase in the concentration of ornithine in serum and liver by Trp administration is due to suppression of the OAT precursor and OAT expression. Therefore, these results suggest that Trp administration induced increased liver ornithine levels by suppressing ornithine catabolism and consequently increased the substrate supply for polyamine synthesis.

ODC catalyzes the first step in the polyamine biosynthetic pathway for Put, which is then converted into the polyamines spermidine and spermine. ODC provides the only established route for de novo Put biosynthesis in mammals. Sidransky et al. reported that oral administration of Trp to overnight-fasted rats increased hepatic ODC activities [24]. On the other hand, we demonstrated that the expression level of ODC protein was not affected by Trp administration (Figure 4). Taken together, these results suggest that the oral administration of Trp induced the activation of ODC without changing the amount of enzyme protein. Thus, both increased levels of substrate for polyamine synthesis and enhanced ODC activity appear to contribute to the Trp-induced increase in polyamine levels.

Polyamines (Put, spermidine, spermine) are essential for cell proliferation [25,26]. The polyamines play an important role in the regulation of global translation, a major role in the regulation of cell growth, and have a critical role in enhancing liver regeneration following partial hepatectomy [27,28]. In particular, spermidine has an essential and unique role in protein synthesis as the precursor of hypusine. Hypusine is an essential component for post-translational modification to activate the initiation factor eIF5A, which is necessary for initiation of protein synthesis [29,30,31]. In addition, many experimental findings indicate that spermidine is specifically required for the initiation of rat liver regeneration. An increase in liver ornithine, Put and spermidine were demonstrated in regenerating liver [32]. Thus, we speculated that the increase in liver polyamine concentration by Trp administration could contribute to the stimulation of hepatic protein synthesis. Therefore, we assessed the effect of the increase of polyamine level on hepatic protein synthesis by oral administration of Put. Indeed, the oral administration of Put enhanced phosphorylation of S6K1 and 4E-BP1 in a dose-dependent manner (Figure 5). These data support our hypothesis that the increase in liver polyamine concentration by Trp administration contributes to the stimulation of hepatic protein synthesis.

In this study, we demonstrated that BCAAs concentrations in the liver were significantly decreased (Table 2), whereas serum concentrations of BCAAs were significantly increased after Trp administration (Table 1). Moreover, 2-DE and immunoblot analysis suggested post-translational modification of BCKDE1α accompanying with acidic pI shift induced by Trp administration (Table 3) (Figure 3). BCKD catalyzes the rate-limiting step in the oxidation of BCAAs. The major mechanism in BCKD regulation is reversible serine phosphorylation of the E1α subunit by branched-chain alpha-keto acid dehydrogenase kinase (BDK) [33]. At low levels of BCAAs, BCKD is inactivated through phosphorylation of its E1α subunit [34]. The differences between the changes in BCAAs concentrations in the liver and in serum can be explained by competitive inhibition of BCAAs transport by Trp. BCAAs and Trp are transported by the L-type amino acid transporter (LAT) system, which is a major route for providing cells with large neutral amino acids, including branched and aromatic amino acids. It is known that increasing the blood concentration of a large neutral amino acid (LNAA) such as Trp, tyrosine, phenylalanine or the BCAAs increases the brain uptake of that LNAA and reduces uptake of the others [35]. Additionally, it was reported that high leucine levels inhibit Trp uptake in isolated liver cells by competing for membrane transport [36]. Therefore, the elevation of serum concentrations of BCAAs is believed to be caused by competitive inhibition of liver uptake of BCAAs by Trp. Since decreased levels of BCAAs inactivates BCKD [34], we speculate the post-translational modification on BCKDE1α accompanying with acidic pI shift was phosphorylation modification due to the decreased liver BCAAs level by Trp administration. However, because there are other post-translational modifications that affect protein pI [37], further investigation is required to conclude about the post-translational modification on BCKDE1α. The first step in the catabolism of BCAAs, reversible transamination, is catalyzed by one of two isoforms of branched-chain amino acid aminotransferase (BCAT), the mitochondrial isoenzyme (BCATm), and the cytosolic isoenzyme (BCATc). Neither BCAT isoenzyme is found in the liver [38]. The liver is therefore unable to perform the first step of BCAAs metabolism, namely, their transamination to the corresponding keto acid. Thus, it would be difficult to assess the physiological effects of phosphorylation of hepatic BCKDE1α by Trp administration. The study of tissues other than the liver are required to explain the mechanism of increase in serum BCAAs levels by Trp administration.

We demonstrated that Trp administration acutely stimulated protein synthesis by increasing of ornithine levels and polyamine synthesis through suppression of OAT expression. The important role of serotonin in liver regeneration was reported previously [9]. It was also reported that Trp induced mTOR activation was mediated by serotonin [10]. However, oral administration of Trp did not affect the liver serotonin concentration (1.2-fold, *p* = 0.378) in the present study. On the other hand, there was a significant increase in the liver kynurenine level (652-fold, *p* < 0.005) after Trp administration. The kynurenine pathway is responsible for major dietary Trp degradation (~95%) and tryptophan 2,3-dioxygenase (TDO) is the rate-limiting enzyme of the kynurenine pathway [39]. The TDO enzymatic activity is regulated by Trp via enhanced conjugation of the apoenzyme with its heme cofactor and stabilization of the holoenzyme. It was reported that the administration of 500 mg/kg of Trp increased the half-life of TDO holoenzyme from 7.7 to 11.4 h [40]. Based on these data, we speculated that the serotonin synthesis was not increased because of the activation of the kynurenine pathway by Trp administration. In addition, it is suggested that the effect of Trp administration-induced acute protein synthesis in this study is independent of the serotonin mediated pathway which was reported previously.

**Figure 6:**
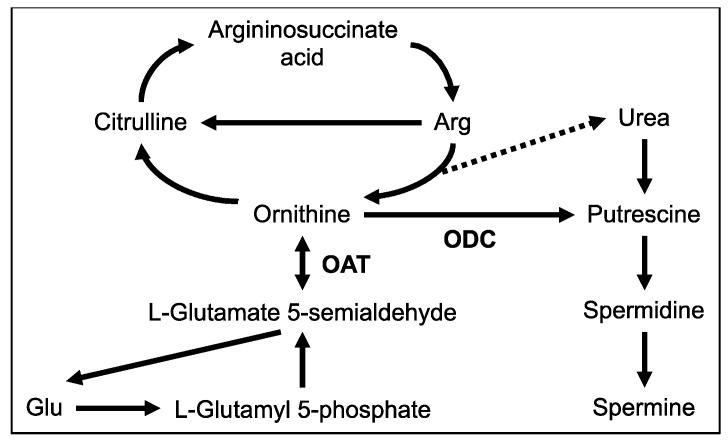
Image of ornithine metabolic pathways in the liver. OAT—ornithine aminotransferase.

In summary, this study was performed in order to identify candidate proteins and metabolites likely to play a role in the stimulation of hepatic protein synthesis by Trp. The expression of OAT, the main catabolic enzyme for ornithine (Figure 6), was significantly decreased by Trp administration. In line with that, the concentration of ornithine was increased in both serum and liver. Additionally, Trp administration significantly increased concentrations of liver polyamines, a metabolic product of ornithine. Finally, we demonstrated enhanced hepatic protein synthesis by oral administration of Put, a primary substrate of other polyamines. Based on these results, we speculate that the increase in ornithine levels through suppression of OAT expression by Trp administration may lead to accelerated polyamine synthesis, thereby promoting protein synthesis in the liver.

## Figures and Tables

**Table 1:** Serum amino acid concentrations in rats administered Tryptophan (Trp).

Amino Acid	Control (nmol/mL)		Trp 1h (nmol/mL)		Trp 3h (nmol/mL)		Ratio vs. Control	
							Trp 1h	Trp 3h
P-Ser	8.78	±1.75	8.73	±0.85	12.26	±1.95	1.0	1.4
Tau	327.62	±18.54 ^a^	523.95	±37.82 ^b^	448.06	±29.40 ^b^	1.6	1.4
Urea	3869.69	±437.71	3710.70	±308.08	3278.09	±260.76	1.0	0.8
Asp	14.87	±1.502 ^a^	100.41	±10.28 ^b^	114.11	±27.90 ^b^	6.8	7.7
Thr	285.51	±10.21 ^a^	370.00	±12.49 ^b^	274.05	±16.69 ^a^	1.3	1.0
Ser	240.41	±2.92	283.73	±8.87	266.28	±19.97	1.2	1.1
Asn	27.81	±1.38	32.35	±1.95	29.46	±2.25	1.2	1.1
Glu	130.72	±13.50 ^a^	238.00	±4.104 ^b^	279.12	±34.58 ^b^	1.8	2.1
Gln	851.87	±49.66 ^a^	1043.51	±32.76 ^ab^	1153.35	±67.22 ^b^	1.2	1.4
Gly	313.06	±10.69 ^a^	247.47	±12.76 ^ab^	177.92	±24.04 ^b^	0.8	0.6
Ala	290.56	±12.28	335.77	±42.77	349.12	±46.00	1.2	1.2
Cit	77.56	±4.20 ^a^	84.75	±4.59 ^a^	107.91	±7.18 ^b^	1.1	1.4
α-ABA	32.88	±1.55 ^a^	39.89	±1.89 ^a^	71.02	±3.81 ^b^	1.2	2.2
Val	143.72	±5.66 ^a^	188.51	±5.49 ^ab^	226.90	±16.36 ^b^	1.3	1.6
Met	49.75	±2.06	49.41	±2.46	50.26	±4.61	1.0	1.0
Cysta	1.35	±0.41	1.49	±0.12	1.67	±0.38	1.1	1.2
Ile	89.33	±3.62 ^a^	113.50	±2.46 ^b^	129.27	±8.06 ^b^	1.3	1.4
Leu	124.44	±5.73 ^a^	156.84	±2.82 ^a^	201.45	±15.59 ^b^	1.3	1.6
Tyr	80.86	±4.65	82.45	±1.93	78.07	±3.16	1.0	1.0
Phe	65.73	±4.01	73.60	±3.06	75.78	±3.11	1.1	1.2
NH3	191.52	±18.68	181.06	±9.92	209.50	±21.01	0.9	1.1
Orn	38.19	±1.99 ^a^	37.63	±2.46 ^a^	79.37	±6.20 ^b^	1.0	2.1
His	45.99	±2.68 ^a^	57.21	±2.25 ^ab^	69.68	±6.58 ^b^	1.2	1.5
Lys	645.80	±28.50 ^a^	424.19	±12.04 ^b^	345.40	±35.18 ^b^	0.7	0.5
3M-His	9.24	±0.47	8.04	±0.52	7.59	±0.66	0.9	0.8
Trp	32.12	±2.62 ^a^	1035.90	±36.86 ^b^	856.38	±113.49 ^b^	32.3	26.7
Arg	140.04	±6.26 ^a^	111.45	±5.13 ^b^	110.89	±6.84 ^b^	0.8	0.8
Hypro	55.26	±2.24 ^a^	46.12	±2.37 ^ab^	37.99	±3.41 ^b^	0.8	0.7
Pro	146.39	±4.55	143.27	±6.11	141.85	±11.30	1.0	2.3

Different letters indicate significant differences between groups (*p* < 0.05). Values represent means ± SEM, *n* = 4–5.

**Table 2:** Changes in the hepatic levels of polyamines, related amino acids, and BCAAs at 3 h after Trp administration.

Metabolite	*m/z*	Migration Time	Relative Area						
			Control		Trp 3h		Trp 3h vs. Control		
			Mean	SEM	Mean	SEM	Ratio	*p*-Value	
Glu	148.060	10.59	8.7 × 10^−2^	4.0 × 10^−3^	2.0 × 10^−1^	4.4 × 10^−3^	2.3	4.4 × 10^−5^	***
*N*-Acetylglutamic acid	188.056	12.75	3.2 × 10^−4^	2.9 × 10^−5^	2.6 × 10^−4^	6.4 × 10^−6^	0.8	0.151	
*N*-Acetylornithine	175.107	9.34	1.5 × 10^−4^	3.8 × 10^−6^	2.8 × 10^−4^	1.6 × 10^−5^	1.8	0.011	*
Ornithine	133.097	6.77	1.3 × 10^−2^	6.9 × 10^−4^	1.8 × 10^−2^	1.4 × 10^−3^	1.5	0.039	*
Citrulline	176.103	10.70	1.8 × 10^−3^	1.7 × 10^−4^	1.5 × 10^−3^	1.8 × 10^−4^	0.8	0.315	
Argininosuccinic acid	291.129	9.23	9.5 × 10^−5^	4.4 × 10^−6^	2.6 × 10^−4^	5.8 × 10^−6^	2.7	4.6 × 10^−5^	***
Arg	175.118	7.06	3.5 × 10^−4^	2.3 × 10^−5^	4.4 × 10^−4^	2.1 × 10^−5^	1.3	0.045	*
Urea	61.040	19.23	4.8 × 10^−2^	2.4 × 10^−3^	4.5 × 10^−2^	7.5 × 10^−4^	0.9	0.309	
Putrescine	89.108	4.74	1.7 × 10^−4^	9.8 × 10^−6^	1.8 × 10^−3^	1.8 × 10^−4^	11	0.012	*
Spermidine	146.165	4.56	2.3 × 10^−3^	1.2 × 10^−4^	4.2 × 10^−3^	2.0 × 10^−4^	1.8	0.003	**
Spermine	203.222	4.50	1.2 × 10^−4^	8.7 × 10^−6^	1.7 × 10^−4^	4.6 × 10^−6^	1.3	0.023	*
Val	118.086	9.78	2.6 × 10^−2^	2.4 × 10^−4^	2.0 × 10^−2^	2.5 × 10^−4^	0.8	8.8 × 10^−5^	***
Leu	132.101	10.04	4.6 × 10^−2^	9.8 × 10^−4^	3.8 × 10^−2^	3.5 × 10^−4^	0.8	0.008	**
Ile	132.101	9.95	2.0 × 10^−2^	1.8 × 10^−4^	1.6 × 10^−2^	3.7 × 10^−5^	0.8	0.001	**
Isobutyryl CoA_divalent	417.570	9.70	N.D.	N.A.	N.D.	N.A.	N.A.	N.A.	

Values represent means ± SEM, *n* = 3. N.D.: Not Detected; N.A.: Not Available. (*), (**), and (***) indicate significant differences between control vs. Trp 3h (*p* < 0.05), (*p* < 0.01), and (*p* < 0.001), respectively. BCAAs—Branched-chain amino acids.

**Table 3:** Protein identification by peptide mass fingerprinting using MALDI-TOF MS.

Spot No.	Protein	Accession No.	Obs. pI	Obs. MM	Calc. pI	Calc. MM	Mascot Score	Protein Seq. Coverage	-Fold Change Trp 1h	-Fold Change Trp 3h
U1	2-oxoisovalerate dehydrogenase subunit alpha, mitochondrial (Fragment)	P11960	5.0	42.5	7.6	50.1	132	37	-	3.14
U2			5.4	42.5			102	30	-	3.00
U3			6.1	41.6			103	34	-	0.28
U4	Ornithine aminotransferase, mitochondrial precursor	P04182	6.6	41.4	6.5	48.3	107	34	0.61	0.13

Obs. MM: Observed molecular mass; Calc. MM: Calculated molecular mass; Seq.: Sequence.

## References

[1] Sainio E.L., Pulkki K., Young S.N. (1996). L-Tryptophan: Biochemical, nutritional and pharmacological aspects. Amino Acids.

[2] Young V.R. (1994). Adult amino acid requirements: The case for a major revision in current recommendations. J. Nutr..

[3] Reilly J.G., McTavish S.F., Young A.H. (1997). Rapid depletion of plasma tryptophan: A review of studies and experimental methodology. J. Psychopharmacol..

[4] Sidransky H., Verney E., Sarma D.S. (1971). Effect of tryptophan on polyribosomes and protein synthesis in liver. Am. J. Clin. Nutr..

[5] Sidransky H., Sarma D.S., Bongiorno M., Verney E. (1968). Effect of dietary tryptophan on hepatic polyribosomes and protein synthesis in fasted mice. J. Biol. Chem..

[6] Kurl R.N., Verney E., Sidransky H. (1988). Identification and immunohistochemical localization of a tryptophan binding protein in nuclear envelopes of rat liver. Arch. Biochem. Biophys..

[7] Sidransky H., Verney E., Cosgrove J.W., Schwartz A.M. (1992). Studies with compounds that compete with tryptophan binding to rat hepatic nuclei. J. Nutr..

[8] Sidransky H., Verney E. (1997). Differences in tryptophan binding to hepatic nuclei of NZBWF1 and Swiss mice: Insight into mechanism of tryptophan’s effects. J. Nutr..

[9] Lesurtel M., Graf R., Aleil B., Walther D.J., Tian Y., Jochum W., Gachet C., Bader M., Clavien P.A. (2006). Platelet-derived serotonin mediates liver regeneration. Science.

[10] Osawa Y., Kanamori H., Seki E., Hoshi M., Ohtaki H., Yasuda Y., Ito H., Suetsugu A., Nagaki M., Moriwaki H. (2011). L-tryptophan-mediated enhancement of susceptibility to nonalcoholic fatty liver disease is dependent on the mammalian target of rapamycin. J. Biol. Chem..

[11] Reeves P.G., Nielsen F.H., Fahey G.C. (1993). AIN-93 purified diets for laboratory rodents: Final report of the American Institute of Nutrition ad hoc writing committee on the reformulation of the AIN-76A rodent diet. J. Nutr..

[12] Anthony J.C., Yoshizawa F., Anthony T.G., Vary T.C., Jefferson L.S., Kimball S.R. (2000). Leucine stimulates translation initiation in skeletal muscle of postabsorptive rats via a rapamycin-sensitive pathway. J. Nutr..

[13] Crozier S.J., Kimball S.R., Emmert S.W., Anthony J.C., Jefferson L.S. (2005). Oral leucine administration stimulates protein synthesis in rat skeletal muscle. J. Nutr..

[14] Yoshizawa F., Mochizuki S., Sugahara K. (2013). Differential dose response of mTOR signaling to oral administration of leucine in skeletal muscle and liver of rats. Biosci. Biotechnol. Biochem..

[15] Kabuyama Y., Langer S.J., Polvinen K., Homma Y., Resing K.A., Ahn N.G. (2006). Functional proteomics identifies protein-tyrosine phosphatase 1B as a target of RhoA signaling. Mol. Cell. Proteom. MCP.

[16] Soga T., Ueno Y., Naraoka H., Ohashi Y., Tomita M., Nishioka T. (2002). Simultaneous determination of anionic intermediates for Bacillus subtilis metabolic pathways by capillary electrophoresis electrospray ionization mass spectrometry. Anal. Chem..

[17] Soga T., Ohashi Y., Ueno Y., Naraoka H., Tomita M., Nishioka T. (2003). Quantitative metabolome analysis using capillary electrophoresis mass spectrometry. J. Proteome Res..

[18] Sugimoto M., Wong D.T., Hirayama A., Soga T., Tomita M. (2010). Capillary electrophoresis mass spectrometry-based saliva metabolomics identified oral, breast and pancreatic cancer-specific profiles. Metab. Off. J. Metab. Soc..

[19] Levillain O., Diaz J.J., Reymond I., Soulet D. (2000). Ornithine metabolism along the female mouse nephron: Localization of ornithine decarboxylase and ornithine aminotransferase. Pflug. Arch. Eur. J. Physiol..

[20] Inana G., Chambers C., Hotta Y., Inouye L., Filpula D., Pulford S., Shiono T. (1989). Point mutation affecting processing of the ornithine aminotransferase precursor protein in gyrate atrophy. J. Biol. Chem..

[21] Brosnan J.T., Brosnan M.E. (2006). Branched-chain amino acids: Enzyme and substrate regulation. J. Nutr..

[22] Paxton R., Kuntz M., Harris R.A. (1986). Phosphorylation sites and inactivation of branched-chain alpha-ketoacid dehydrogenase isolated from rat heart, bovine kidney, and rabbit liver, kidney, heart, brain, and skeletal muscle. Arch. Biochem. Biophys..

[23] Kong X., Wang X., Yin Y., Li X., Gao H., Bazer F.W., Wu G. (2014). Putrescine stimulates the mTOR signaling pathway and protein synthesis in porcine trophectoderm cells. Biol. Reprod..

[24] Sidransky H., Murty C.N., Myers E., Verney E. (1983). Tryptophan-induced stimulation of hepatic ornithine decarboxylase activity in the rat. Exp. Mol. Pathol..

[25] Pegg A.E., Casero R.A. (2011). Current status of the polyamine research field. Methods Mol. Biol..

[26] Pegg A.E., McCann P.P. (1982). Polyamine metabolism and function. Am. J. Physiol..

[27] Alhonen L., Rasanen T.L., Sinervirta R., Parkkinen J.J., Korhonen V.P., Pietila M., Janne J. (2002). Polyamines are required for the initiation of rat liver regeneration. Biochem. J..

[28] Mandal S., Mandal A., Johansson H.E., Orjalo A.V., Park M.H. (2013). Depletion of cellular polyamines, spermidine and spermine, causes a total arrest in translation and growth in mammalian cells. Proc. Natl. Acad. Sci. USA.

[29] Schnier J., Schwelberger H.G., Smit-McBride Z., Kang H.A., Hershey J.W. (1991). Translation initiation factor 5A and its hypusine modification are essential for cell viability in the yeast Saccharomyces cerevisiae. Mol. Cell. Biol..

[30] Chattopadhyay M.K., Park M.H., Tabor H. (2008). Hypusine modification for growth is the major function of spermidine in Saccharomyces cerevisiae polyamine auxotrophs grown in limiting spermidine. Pro. Natl. Acad. Sci. USA.

[31] Park M.H., Nishimura K., Zanelli C.F., Valentini S.R. (2010). Functional significance of eIF5A and its hypusine modification in eukaryotes. Amino Acids.

[32] Jung Y.S., Kim S.J., Kwon do Y., Kim Y.C. (2012). Metabolomic analysis of sulfur-containing substances and polyamines in regenerating rat liver. Amino Acids.

[33] Popov K.M., Zhao Y., Shimomura Y., Kuntz M.J., Harris R.A. (1992). Branched-chain alpha-ketoacid dehydrogenase kinase.Molecular cloning, expression, and sequence similarity with histidine protein kinases. J. Biol. Chem..

[34] Murakami T., Matsuo M., Shimizu A., Shimomura Y. (2005). Dissociation of branched-chain alpha-keto acid dehydrogenase kinase (BDK) from branched-chain alpha-keto acid dehydrogenase complex (BCKDC) by BDK inhibitors. J. Nutr. Sci. Vitaminol..

[35] Fernstrom J.D. (2013). Large neutral amino acids: Dietary effects on brain neurochemistry and function. Amino Acids.

[36] Salter M., Bender D.A., Pogson C.I. (1985). Leucine and tryptophan metabolism in rats. Biochem. J..

[37] Zhu K., Zhao J., Lubman M.D., Miller M.R., Barder J.T. (2005). Protein pI Shifts due to Posttranslational Modifications in the Separation and Characterization of Proteins. Anal. Chem..

[38] Hutson S.M., Wallin R., Hall T.R. (1992). Identification of mitochondrial branched chain aminotransferase and its isoforms in rat tissues. J. Biol. Chem..

[39] Badawy A.A. (2017). Kynurenine Pathway of Tryptophan Metabolism: Regulatory and Functional Aspects. Int. J. Tryptophan Res..

[40] Badawy A.A., Evans M. (1975). Regulation of rat liver tryptophan pyrrolase by its cofactor haem: Experiments with haematin and 5-aminolaevulinate and comparison with the substrate and hormonal mechanisms. Biochem. J..

